# Health utility of patients with established rheumatoid arthritis and its influencing factors: a multi-center study in China

**DOI:** 10.1038/s41598-024-64772-4

**Published:** 2024-06-19

**Authors:** Chuchuan Wan, Yuankai Huang, Qiqi Wang, Pei Wang, Xiaoyu Xi

**Affiliations:** 1 The Research Center of National Drug Policy & Ecosystem, China Pharmaceutical University, No. 639 Longmian Avenue, Jiangning District, Nanjing City, Jiangsu Province China; 2 School of Public Health, Fudan University, No. 130 Dongan Road, Shanghai, China; 3 Key Lab of Health Technology Assessment, National Health Commission of the People’s Republic of China (Fudan University), Shanghai, China

**Keywords:** Rheumatoid arthritis, Disease prevention, Quality of life, Rehabilitation

## Abstract

To assess the health utility value (HUV) of Rheumatoid Arthritis (RA) patients and its influencing factors in China. A cross-sectional survey was conducted in 8 tertiary hospitals across four capital-cities. The demographic characteristics, patient-reported outcomes including the HUV got by EQ-5D-5L, clinical characteristics, and clinician-reported outcomes of 171 RA patients were collected both from themselves and their physicians. Both the univariate and multivariate analyses were used to assess the potential factors of EQ-5D-5L HUV of the patients. The mean age of the patients was 50.7 years, with female being 64.9% (n = 111). The mean HUV and EQ visual analogue scale score of all patients were 0.586 and 47.3, respectively. The univariate analysis showed that the patients who were female, older, living in rural areas, with lower education level, advanced disease stage, higher the patient's assessment of arthritis pain visual analogue scale (PtAAP-VAS), the patient's global assessment of disease activity visual analogue scale (PtGADA-VAS), and the Physician’s global assessment of disease activity visual analogue scale (PhGADA-VAS) scores had significantly lower EQ-5D-5L HUVs. The multivariate analysis further suggested that older age, female, higher body mass index and higher PtGADA-VAS score were statistically significantly related to lower HUVs. The study provided the HUVs for RA patients with different characteristics and outcomes, which could be used in the economic evaluation of interventions for the RA patients. The identified factors could also assist the health care managing and improving the health-related quality of life on RA patients.

## Introduction

Rheumatoid Arthritis (RA) which is a chronic autoimmune disorder is symmetrical, polyarticular, erosive, recurrent and incurable, mostly occurring in joints such as hands, feet, elbows, shoulders, ankles and knees, etc.^1,2^. Globally, its current prevalence is about 0.1 ~ 1%^2–4^. While it is 0.28 ~ 0.42% for China^1,3,4^, thus affecting about 5 million patients with the male female ratio being 1:4^5,6^. Most patients are middle-aged when diagnosed and the peak is 50 years old^7^. And another important fact is that most early RA patients have no typical clinical symptoms^8,9^. The data of Chinese Registry of rheumatoid arthritis (CREDIT)^7^ shows that the proportion of RA patients at middle and advanced stages accounts for about 82% (disease stage based on the degree of joint destruction^10^, including I/early, II/middle, III ~ IV/advanced). Thus, the treatment and economic evaluation mainly occur in these patients^11,12^. These reduce health-related quality of life (HRQOL) on RA patients, and increase the pressure on the medical expenditure. *The 2019 annual report of China's rheumatoid arthritis* shows that the annual economic burden of RA patients was as high as 72 million Chinese Yuan (CNY) (10.44 million United States Dollar (USD), converted using 2019 annual exchange rate of CNY to USD). When the influence of per capita disability adjusted life years (DALYs) was included, the annual economic burden was as high as 902 million CNY (130.75 million USD) and per capita annual economic burden was 15,718 CNY (2,278.48 USD)^7^.

Given the severe disease and economic burden of RA and finite health care resources, it is important to assess the cost-effectiveness of health interventions/drugs treating RA. Cost-utility analysis (CUA) is the most widely used economic evaluation method^13^, in which quality adjusted life years (QALYs) is adopted as the main health outcome^14^. To calculate QALYs, health utility value (HUV) is required as the quality of life weight adjusting life expectancy. HUV is an indicator reflecting people's preferences for specific health states and represents a certain orientation of societal or individual values. In the world, a number of studies have measured the HUV on RA patients using utility instruments such as EQ-5D, SF-6D and HUI^12,15–24^, and believe that age, gender and disease severity etc. may be associated with HUV of RA patients. Four studies have also provided the HUVs for the patients by using the EQ-5D-3L^12,23^, EQ-5D-5L^24^ and SF-6D^25^ in China. However, those studies were based on either single center^12,23^ or two centers^24^; and did not report the HUVs for the patients at different disease stages and identify the factors affecting HUVs.

Hence, the study aims to assess the HUVs of RA patients at middle and advanced stages and the influencing factors using a multi-center cross-sectional data in China. It is benefit for conducting the economic evaluations of interventions for the RA patients and managing the RA disease, which in turn reduces the burden of RA on patients and society.

## Method

Cross-sectional data of RA patients at middle and advanced stages from 8 tertiary hospitals across four provincial capitals Nanjing, Hangzhou, Chengdu, and Shijiazhuang (two of each) were adopted, in which Chinese version of the EQ-5D-5L was used to measure and value HRQOL. The patients were enrolled by trained investigators based on quota sampling during June to July 2020.

### Study participants

Based on available resources and rules of thumb, a total of 200 patients (50 in each city, male to female = 1:2) were planned to recruit. The inclusion criteria were: (1) Informed and voluntary; (2) 18–70 years (Considering the challenges and precision involved in questionnaire completion by older adults, previous research protocols^26^, and available resources); (3) Diagnosed with RA according to the 2010 American College of Rheumatology (ACR)/European League Against Rheumatism (EULAR) classification diagnostic criteria (score ≥ 6)^27^. The exclusion criteria were: (1) Pregnant women, individuals with psychiatric disease, and patients who were unconscious and unable to feedback their condition; (2) Patients suffering from other serious diseases such as tumors, myocardial infarct.

### Data collection

A total of 16 interviewers who were trained and divided into 8 groups were responsible for the data collection in each of the 8 hospitals. In the corresponding departments of each hospital during the survey (no distinction between outpatient and inpatient), the interviewers introduced the purpose and content of the research to the patients and their attending physicians and provided written informed consent to them. After that, consenting patients and physicians were asked to complete the respective questionnaires independently in a quiet room. When patients have any questions about the questionnaire, interviewers must provide explanations, but should not guide or actively interfere with the patients' completion of the questionnaire. The completed questionnaires were reviewed by the interviewers to identify any obvious errors or blanks. After the questionnaires were retrieved, the data would be digitalized and reviewed by 2 independent and transparent auditors.

### Questionnaires for the patients and physicians

Two respective questionnaires for the patients and physicians were preliminarily designed according to the literature^15,28^. Then, based on the opinions of experts, we revised the questionnaires and conducted a pilot survey in 2 tertiary hospitals of Nanjing to validate the rationality, readability and comprehensibility of questionnaires (Considering that the subjective questions included in the questionnaire are all from validated scales, we did not repeat the validation of the psychological measurement properties of the questionnaire.) The questionnaires were revised and formed the final version based on the results of pilot survey and the suggestions from experts according the results of pilot survey. The rationality, readability and comprehensibility of the questionnaire had been affirmed by the experts and supported by the pilot survey.

The questionnaire for the patients consists of two parts. Part one collected patients’ HRQOL measured by Chinese version of the EQ-5D-5L and other self-reported outcome measures including general health, arthritis pain and disease activity. General health was evaluated by using a four-level scale: good, general, bad and very bad. Arthritis pain and disease activity were assessed using two visual analogue scales (VAS) respectively. The patient's assessment of arthritis pain VAS (PtAAP-VAS)^29^ measured the degree of pain they were experiencing on the day of survey: 0 means no pain, 100 means the most severe pain. The patient's global assessment of disease activity VAS (PtGADA-VAS)^30,31^ assessed the current disease activity: 0 means that the patients feel very well and there are no symptoms, 100 means that the patients feel very bad and there are severe symptoms. Part two used questions assessing patients’ demographic characteristics including gender, age, ethnicity, height, weight, region, marital status, education level, occupation, types of medical insurance and personal annual income.

The questionnaire for the physicians assessed the patients’ clinical characteristics and clinician-reported outcomes including disease stage (based on the X-ray examination results of joints, Grade I /early stage: no bone destructive changes; Grade II/middle stage: osteoporosis with mild cartilage damage; Grade III ~ IV/advanced stage: osteoporosis with cartilage or bone destruction and joint deformity)^32^, treatment modalities (inpatient/outpatient), erythrocyte sedimentation rate (ESR) (unit: mm/h), high-sensitivity C-reactive protein (CRP) (unit: mg/L), symptoms and functional ability, swollen joints count (SJC) and tender joints count (TJC). Among them, the symptoms and functional ability was assessed using the Physician’s global assessment of disease activity VAS (PhGADA-VAS)^30,31^ (0 and 100 mean the patients are asymptomatic and normal activities are not restricted, and have severe symptoms which cannot be tolerated and do not have the ability to carry out the normal activities, respectively). SJC, TJC, CRP and ESR were used to calculate the 28 joint counts (DAS28) scores, including DAS28-CRP score and DAS28-ESR score^33^. Higher DAS28 scores indicates higher disease activity. Disease activity can be divided into four states, including remission (DAS28 scores < 2.6), low activity group (2.6 ≤ DAS28 scores < 3.2), moderate activity group (3.2 ≤ DAS28 scores < 5.1), and high activity group (DAS28 scores ≥ 5.1)^33^.

### EQ-5D-5L

Compared with EQ-5D-3L, the EQ-5D-5L is improved measurement properties in terms of its health-state descriptive system and HUV^34^; and its reliability and validity have been verified in China^35^. Its descriptive system assesses the subjects’ health status on the day of survey on five dimensions (mobility, self-care, usual activities, pain/discomfort, and anxiety/depression), each of which uses five severity levels (no problems, slight problems, moderate problems, severe problems, and unable to/extreme problems) and produces a total of 3125 (5^5^) health states^34^. Each health state can be expressed using a 5-digit number. For example, “12,345” means no problems in mobility, slight problems in self-care, moderate problems in usual activities, severely pain/discomfort and extremely anxious/depressed. All the health states defined by the system can be converted into HUVs using a value set. In this study, Chinese version of the EQ-5D-5L and the value set^36^ for China was adopted. The EQ-5D-5L also includes the EQ visual analogue scale (EQ-VAS) assesses the self-reported health status of subjects through a straight line (0: the worst health you can imagine; 100: the best health you can imagine)^34^.

### Data analysis

#### Descriptive statistics

Descriptive statistics such as mean and standard deviation (SD) for continuous variables, frequency and percentage for categorical variables were used to depict the characteristics and outcomes of the patients. The distribution of EQ-5D-5L data (i.e., responses on each dimension, HUV and VAS score) of the patients with different characteristics were presented. The skewness and kurtosis of EQ-5D-5L HUV were also showed. We also compared the average age (*p* > 0.1) and gender ratio (*p* < 0.01) of the patients with that of Chinese RA patients^15,37,38^, and weighted the EQ-5D-5L data according to the gender ratio of Chinese RA patients^37^.

#### Univariate analysis

In order to identify significant factors of the patients’ HUV, Kruskal–Wallis test or univariate regression were performed on the categorical variables or continuous variables, respectively. The variables included patient's characteristics and patient-/clinician-reported outcomes, of which age, body mass index (BMI) and person's annual income were processed as categorical variables. Kruskal–Wallis test was also performed on patients’ responses on EQ-5D-5L.

#### Multivariate analysis

**Figure 1:**
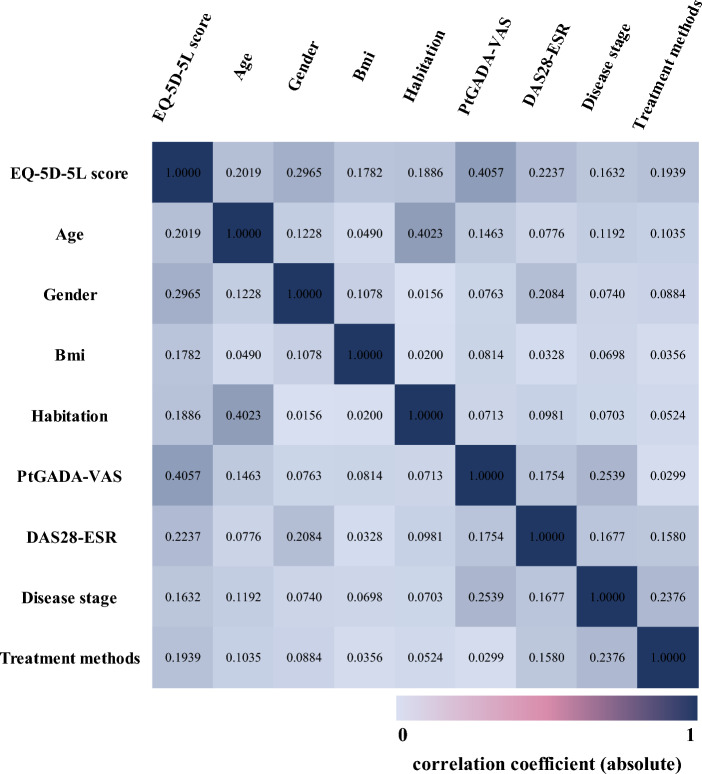
The correlation heatmap of variables.

Beta model was used for the multivariate regression analysis because of the characteristics of the distribution of HUV (i.e., non-normal distributed and censored at (1). Beta model required the value of the dependent variable between 0–1. The EQ-5D-5L scores were adjusted through the formula: adjusted score = (original score + 0.391)/1.391 (The range of EQ-5D-5L score was − 0.391 to 1 based on the Chinese value set. If the original score was − 0.391 or 1, the adjusted score was added or subtracted e^−12^ to ensure it fell between 0 and 1.) Given the gender ratio in sample was different from that of Chinese RA patients, we used the gender weighting in the regression. The dependent variable was the EQ-5D-5L HUV. Refereeing the recommendations of clinical experts, literature^15–17^, and the results of univariate analysis, some of the characteristics and outcomes were included as explanatory variables in the regression analysis. In order to reduce multicollinearity among the variables, Spearman rank correlations were used to test the correlations between them (correlation coefficients: very weak = 0–0.19; weak = 0.20–0.39; moderate = 0.40–0.59; strong = 0.60–0.79; and very strong = 0.80–1.00). For a pair of variables with the coefficient higher than 0.4, the variable that was also correlated with other variables more would be excluded (Supplementary material Appendix 1). The variables entered into the final model are related to the EQ-5D-5L score, but the correlation between these variables is low, including demographic variables age, gender, BMI and habitation; and clinical variables PtGADA-VAS, DAS28-ESR, disease stage and treatment modalities (the correlation heatmap is shown in Fig. 1). Finally, we would measure the multicollinearity in regression model through variance inflation factor (VIF).

All above analysis were performed on Microsoft® Excel 2021 and stata15.

### Ethics approval and consent to participate

This study was conducted in accordance with the principles of the Declaration of Helsinki and approved by the Clinical Trial Ethics Committee. Clinical Trial Ethics Committee of Huashan Hospital Affiliated to Fudan University (Reference Number 2019–252). Written informed consent to participate was signed by all participators.

## Result

### Sociodemographic characteristics and patient-reported outcomes

A total of 171 eligible patients were included in the analysis (Table 1). Their mean age (SD) was 50.7 (12.0) years, which was not significantly different from that of the general Chinese RA patient population (*p* = 0.31)^15,37,38^. On the other hand, the proportion of female (64.9%) was significantly lower than that (80%) of the population (*p* = 0.00)^37^. The majority of patients were living in rural area (56.1%) and perceived their health status as general (76.6%). The mean of PtAAP-VAS and PtGADA-VAS scores (SD) were 63.9 (18.2) and 64.5 (19.2), respectively.

**Table 1:** Sociodemographic characteristics, patient-reported outcomes and associated EQ-5D-5L utility values of RA patients.

	n (%)/Mean (SD)	Mean HUV (SD)/CHUV	*p* Value	95% CI	
Characteristics					
Total	171(100.00%)	0.586(0.279)		0.544	0.629
Gender			0.000**		
Male	60(35.09%)	0.697(0.233)		0.637	0.758
Female	111(64.91%)	0.527(0.285)		0.473	0.580
Age (years)			0.001**		
18–39	32(18.71%)	0.738(0.227)		0.656	0.819
40–49	35(20.47%)	0.596(0.234)		0.516	0.677
50–59	58(33.92%)	0.513(0.284)		0.438	0.587
60–70	46(26.90%)	0.567(0.304)		0.476	0.657
Ethnicity			0.369		
Han	162(94.74%)	0.582(0.282)		0.538	0.625
Other	9(5.26%)	0.673(0.212)		0.510	0.836
BMI			0.061		
BMI < 18.5	19(11.11%)	0.700(0.185)		0.611	0.789
18.5 ≤ BMI < 24	95(55.56%)	0.601(0.274)		0.545	0.657
24 ≤ BMI	57(33.33%)	0.525(0.302)		0.445	0.605
Habitation			0.016*		
Urban	75(43.86%)	0.639(0.274)		0.576	0.703
Rural	96(56.14%)	0.545(0.278)		0.489	0.601
Marriage			0.026*		
Unmarried	12(7.02%)	0.740(0.198)		0.614	0.865
Married	94(54.97%)	0.606(0.293)		0.546	0.666
Divorce/Widowed	13(7.60%)	0.571(0.282)		0.401	0.742
Not reported	52(30.41%)	0.520(0.256)		0.449	0.592
Occupation			0.170		
Farmer	96(56.14%)	0.547(0.280)		0.491	0.604
Worker	40(23.39%)	0.648(0.280)		0.558	0.737
Government-affiliated institutions	7(4.09%)	0.727(0.198)		0.543	0.910
Retiree	7(4.09%)	0.525(0.396)		0.159	0.891
Other	21(12.28%)	0.623(0.235)		0.516	0.730
Education			0.001**		
Primary school or below	74(43.27%)	0.508(0.290)		0.441	0.575
Middle school	67(39.18%)	0.608(0.263)		0.544	0.672
Undergraduate or above	30(17.54%)	0.732(0.224)		0.648	0.815
Person's annual income			0.228		
(0,30,000)	31(18.13%)	0.556(0.280)		0.453	0.659
[30000,60,000)	17(9.94%)	0.675(0.284)		0.529	0.821
≥ 60,000	16(9.36%)	0.647(0.300)		0.487	0.807
Not reported	107(62.57%)	0.572(0.275)		0.519	0.625
Medical insurance type			0.010**		
BMIUE	46(26.90%)	0.661(0.298)		0.572	0.749
BMIURR	73(42.69%)	0.587(0.275)		0.523	0.651
Not reported	52(30.41%)	0.520(0.256)		0.449	0.592
General health			0.000**		
Good	3(1.75%)	0.781(0.099)		0.534	1.028
General	131(76.61%)	0.634(0.261)		0.589	0.679
Bad	26(15.20%)	0.449(0.287)		0.333	0.565
Very bad	11(6.43%)	0.294(0.219)		0.147	0.441
Patient-reported outcomes					
PtAAP-VAS	–	− 0.006	0.000**	− 0.008	− 0.004
PtGADA-VAS	64.5(19.23)	− 0.006	0.000**	− 0.008	− 0.004

*p* value is used to indicate whether there is a difference in mean utility among different populations under this characteristic, *p < 0.05, **p < 0.01; SD: standard deviation; CHUV: coefficient of health utility value; CI: Confidence Interval; BMIUE: Basic medical insurance for urban employees; BMIURR: Basic medical insurance for urban and rural residents; PtAAP-VAS: patient's assessment of arthritis pain; PtGADA-VAS: patient's global assessment of disease activity.

### Clinical characteristics and clinician-reported outcomes

Clinical characteristics and clinician-reported outcomes were presented in Table 2 and Supplementary material Appendix 2. 146 patients (85.4%) and 25 patients (14.6%) were at middle and advanced disease stages, respectively. Some patients (20 ~ 30%) were at moderate disease activity and most patients (60 ~ 70%) were at high disease activity.

**Table 2:** Clinical characteristics, clinician-reported outcomes and associated EQ-5D-5L utility values of RA patients.

	n (%)/Mean (SD)	Mean HUV (SD)/CHUV	*p* value	95% CI	
Characteristics					
Disease stage			0.033*		
Middle	146(85.38%)	0.612(0.259)		0.568	0.654
Advanced	25(14.62%)	0.440(0.350)		0.295	0.584
Das28-CRP			0.658		
Remission	3(1.75%)	0.744(0.026)		0.679	0.808
Low	3(1.75%)	0.556(0.417)		− 0.480	1.591
Moderate	54(31.58%)	0.606(0.281)		0.529	0.683
High	111(64.91%)	0.574(0.280)		0.521	0.626
Das28-ESR			0.151		
Remission	4(2.34%)	0.766(0.127)		0.564	0.967
Low	6(3.51%)	0.589(0.333)		0.239	0.938
Moderate	41(23.98%)	0.658(0.245)		0.581	0.736
High	120(70.18%)	0.556(0.287)		0.504	0.608
Treatment methods			0.023*		
Hospitalization	16(9.36%)	0.449(0.246)		0.318	0.580
Outpatient	155(90.64%)	0.601(0.279)		0.556	0.645
Clinician-reported Outcomes					
ESR	48.9(29.26)	− 0.003	0.000**	− 0.004	− 0.001
CRP	30.6(36.40)	− 0.001	0.138	− 0.002	0.000
SJC	14.4(9.12)	− 0.004	0.067	− 0.009	0.000
TJC	23.5(14.53)	− 0.002	0.113	− 0.005	0.001
DAS28-ESR	5.6(1.36)	− 0.048	0.002**	− 0.079	− 0.018
DAS28-CRP	5.5(1.20)	− 0.045	0.011*	− 0.080	− 0.010
PhGADA-VAS	63.0(14.22)	− 0.005	0.000**	− 0.008	− 0.002

*p* value is used to indicate whether there is a difference in mean utility among different populations under this characteristic, *p < 0.05, **p < 0.01; SD: standard deviation; CHUV: coefficient of health utility value; CI: Confidence Interval; PhGADA-VAS: physician’s global assessment of disease activity; ESR: erythrocyte sedimentation rate (unit: mm/h); CRP: high-sensitivity C-reactive protein (unit: mg/L); SJC: swollen joints count; TJC: tender joints count; DAS28: disease activity scores including 28 joint counts.

### EQ-5D-5L data

The prevalence of reporting problems on EQ-5D-5L dimensions was showed in Supplementary material Appendix 3. For each dimension, the majority of the patients experienced difficulties, ranging from anxiety/depression (64.3%) to pain/discomfort (84.2%). We also identified several of characteristics (e.g., female, older, lower education level) significantly associated with reporting EQ-5D-5L problems on one-or-more dimension(s) (Supplementary material Appendix 3).

**Figure 2:**
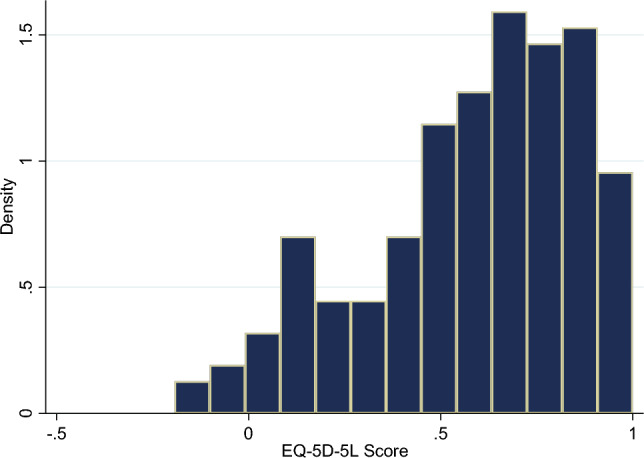
Distribution of the EQ-5D-5L utility Score of RA patients.

The EQ-5D-5L HUVs of the patients presented a left-skewed distribution (skewness: − 0.711; kurtosis: 2.701; see Fig. 2). The mean HUVs (SD) of patients were 0.586 (0.279)/0.561 (adjusted). The HUVs of patients with different characteristics were shown in Tables 1 and 2. Male had higher HUVs than female (0.697 vs. 0.527) and the patients at middle stage had higher HUV (0.611 vs. 0.440) (Table 2). Overall, the higher the disease activity of patients, the lower the HUV (Table 2). The mean EQ-VAS scores of patients were 47.3 (19.6)/47.3 (adjusted). Male and the patients at middle stage also had higher EQ-VAS scores than their counterparts (Supplementary material Appendix 4).

### Univariate analysis of EQ-5D-5L health utility value

The patients with the following characteristics had significantly lower HUVs: female (*p* < 0.01), older (*p* < 0.01), rural areas (*p* < 0.05), divorce/widowed (*p* < 0.05), lower education level (*p* < 0.01), participating in the BMIURR (*p* < 0.01), poor general health (*p* < 0.01), advanced disease stage (*p* < 0.05), hospitalized (*p* < 0.05), higher PtAAP-VAS, PtGADA-VAS, and PhGADA-VAS (*p* < 0.01 for all), higher ESR and SJC (*p* < 0.01 for both) higher DAS28-ESR and DAS28-CRP (*p* < 0.05 for both). The full results were shown in Tables 1 and 2 and Supplementary material Appendix 2.

### Multivariate analysis of EQ-5D-5L health utility value

The results of Beta regression analysis were shown in Table 3. Overall, older age, female and higher BMI were statistically significantly associated with lower EQ-5D-5L HUVs. The patients with advanced disease stage or higher PtGADA-VAS score also had significantly lower HUV. And VIF (Appendix 5) showed there was no multicollinearity between the variables included in the regression model.

**Table 3:** Beta regression analysis of EQ-5D-5L utility value of RA patients.

Variable	Coefficient	SE	*p* value	95% CI	
Age (years)					
18–39	Reference				
40–49	− 0.618	0.157	0.000**	− 0.926	− 0.311
50–59	− 0.626	0.157	0.000**	− 0.935	− 0.317
60–70	− 0.513	0.163	0.002**	− 0.833	− 0.193
Gender					
Male	Reference				
Female	− 0.869	0.122	0.000**	− 1.107	− 0.630
BMI					
BMI < 18.5	Reference				
18.5 ≤ BMI < 24	− 0.455	0.153	0.003**	− 0.755	− 0.155
24 ≤ BMI	− 0.570	0.163	0.000**	− 0.888	− 0.251
Habitation					
Urban	Reference				
Rural	− 0.119	0.104	0.252	− 0.322	0.084
Disease stage					
Middle	Reference				
Advanced	− 0.273	0.136	0.044*	− 0.539	− 0.007
Treatment methods					
Hospitalization	Reference				
Outpatient	0.364	0.166	0.028*	0.039	0.689
PtGADA-VAS	− 0.020	0.003	0.000**	− 0.025	− 0.014
DAS28-ESR	− 0.003	0.035	0.939	− 0.072	0.067
Constant	3.499	0.352	0.000**	2.809	4.189

*p < 0.05, **p < 0.01; SE: Standard error; CI: Confidence interval; PtGADA-VAS: The score of visual analogue scale for patients self-assessing current feelings of disease; DAS28-ESR: Disease Activity Scores including 28 joint counts- erythrocyte sedimentation rate.

## Discussion

As far as we know, previous studies^16,39^ had focused less on the health of RA patients with different disease stages but more on disease activity and no research reported HUV of Chinese patients at different disease stages. Our study was the first study assessing the HUV and its influencing factors of Chinese adult RA patients at different disease stages. Moreover, the study sample came from multiple centers in different regions of China, thus indicating better representativeness. Hence, our findings could be given priority for use in the economic evaluations of health interventions targeting RA patients and was useful for disease management of RA patients in China or other countries which development is similar with China.

Given the proportion of male in sample was significantly larger than that of Chinese RA patients^37^ (*p* < 0.05), we adjusted the results using gender weighting (unadjusted HUV: 0.586; adjusted HUV: 0.561). But both the unadjusted and adjusted HUVs were not only significantly lower than that of the general Chinese population^40,41^ (*p* < 0.01), but also generally lower than that of RA patients provided by other studies in China (0.700, 0.500, 0.660, and 0.652)^12,23–25^. This may be due to the different HUV between the RA patients with different disease stage, as well as the fact that those studies included RA patients at early disease stage. Unfortunately, they did not report HUV of patients with different disease stage or the disutility values of influencing factors. Considering that degree of joint destruction was different with different disease stages, the usefulness of their findings in economic evaluations might be limited. Our study reported the HUV of RA patients at different disease stage or disease activity. As expected, the RA patients at serious stage had substantially lower HUVs than the patients at middle stage (0.612 vs. 0.440). This was consistent with the reality that RA patients at serious stage suffer more distress from illness. Although the RA patients with lower disease activity had higher HUV, we found there were no significant difference in HUV between patients with different disease activity. The reason may that there be patients who were previously disabled due to high disease activity. Post-treatment, their disease was controlled, and they entered a state of low disease activity. However, the patients already had a disability, and the HUV lost due to the disability cannot be regained. Of course, a small sample size may also result in no significant difference in HUV among patients with different disease activity. In addition, we found that nearly 3% of patients have health utility values less than 0, which means they feel that being alive is worse than being dead. Previous studies seem to have paid less attention to these patients. Undoubtedly, these patients were suffering greatly, and we should give them more supports.

Similarly, the HUV, either adjusted or unadjusted, in our study were lower than that of most Asian countries according to a meta analysis (i.e., the mean EQ-5D HUV is 0.66)^42^. Apart from other factors such as ethnicity, cultural and social development, the finding again could be the absence of early RA patients in our sample. Furthermore, studies in Asia had provided disutility values of certain characteristics and outcomes (e.g., higher DAS28 score, female^19,39^, older age^12,19,39,42^ and higher BMI^19,42^). Our results not only included the values of the characteristics, but further provided the values of some rarely measured characteristics such as serious disease stage and hospitalization, thus enriching the data for economic evaluations and pointing to priorities for health management.

From different dimensions measured by EQ-5D-5L, we found that the patients were more likely to be inflicted in functioning dimensions, like mobility (80.91%), usual activities (81.02%). The finding was similar with the results from South Korea^16^. Meanwhile, the proportion of reporting problems in somatic symptom dimensions still reached a height, like pain/discomfort (83.64%) and anxiety/depression (65.33%). Hence, the health care to the RA patients should be comprehensive, considering both physical and psychological aspects.

In terms of the factors of HUV, both the univariate analysis and multivariate analyses showed that sociodemographic characteristics female and older age are related to lower HUV, which were consistent with previous findings in Asia^17,19,39^. The univariate analysis also showed that the HUV of patients in rural areas or received lower education were lower. The former may be due to the social development level: lower social development suggests worse accessibility to healthcare, lower quality of health services, and thus poorer health status. The effect of education on HUV was also detected in south Korea^14^. Another important finding was that the HUV of obese patients was significantly lower than that of others. Obese RA patients reported more problems on mobility, pain/discomfort and anxiety/depression, which may be related to the more serious joint burden of obese patients^43–45^. The finding is in general similar with previous results; while only one study showed an inverse relationship between BMI and HUV^46^. As for the effect of disease stage, both the univariate and multivariate analyses showed an significant adverse effect on HUV. The patients at advanced stage reported more problems on all EQ-5D dimensions except of pain/discomfort dimension. It was surprising to some extend that the patients at middle stage had more problems on pain/discomfort. We could not explain the phenomenon, which needed be further explored by using a larger sample. In terms of the patient-/clinician-reported outcomes, the univariate analysis showed a number of scores (i.e., PtAAP-VAS, PtGADA-VAS, PhGADA-VAS, ESR, SJC, DAS28-ESR and DAS28-CRP) were related to HUVs. The results of multivariate analysis showed that Higher PtGADA-VAS score was more significant association with lower HUVs compared to DAS28-ESR score, which may be due to the PtGADA-VAS score reflected the global influence of the disease on patients’ health.

The study had several limitations that should be noted. First, the cross-sectional design makes impossible to make causal inferences between the EQ-5D-5L HUV and the factors. We were only able to identify factors that may be associated with the HUV of RA patients. More follow-up data were needed if we were to confirm whether these factors lead to changes in HUV of RA patients. Second, the sample size was moderate and the number of patients at advanced disease stage was small, which might have skewed the results. Third, we did not observe the HUV of RA patients at early disease stage, which was influenced by the sample size and RA disease characteristics (RA was difficult to detect in early stages). Fourth, due to the consideration of privacy, some patients were unwilling to disclose their income and insurance type. To maximize the sample size, we did not exclude these patients. However, this led to a significant number of missing observations for variables such as income and type of insurance, potentially introducing bias in identifying the factors influencing HUV in RA patients.

## Conclusion

We provided the EQ-5D-5L utility values of RA patients with various characteristics and outcomes in China, which are useful for the assessment of cost-effectiveness of health interventions for the RA patients. According to our results, more attention could be pay to the patients who are older, female, and obese, as well as with high PtGADA-VAS score, in disease management.

### Supplementary Information

Supplementary Information.

## Data Availability

The datasets generated and/or analyzed during the current study are not publicly available, but they are available from the corresponding author on reasonable request.

## Abbreviations

RA: Rheumatoid Arthritis
HRQOL: Health-related quality of life
CNY: Chinese Yuan
HUV: Health utility value
QALYs: Quality adjusted life years
VAS: Visual analogue scales
PtAAP: The patient's assessment of arthritis pain
PtGADA: The patient's global assessment of disease activity
ESR: Erythrocyte sedimentation rate
CRP: C-reactive protein
SJC: Swollen joints count
TJC: Tender joints count
PhGADA: The Physician’s global assessment of disease activity
DAS28: The 28 joint counts
BMI: Body mass index
SD: Standard deviation
CHUV: Coefficient of health utility value
CI: Confidence Interval
BMIUE: Basic medical insurance for urban employees
BMIURR: Basic medical insurance for urban and rural residents
SE: Standard error
