# Supplementary material for: Health utility of patients with established rheumatoid arthritis and its influencing factors: a multi-center study in China
Source: Sci Rep. 2024 Jun 19;14:14129. doi: 10.1038/s41598-024-64772-4 (PMC11187111; doi:10.1038/s41598-024-64772-4)
Supplement: Supplementary file 1 — Supplementary Information. [file 41598_2024_64772_MOESM1_ESM.pdf]

## Appendix

### Appendix 1 correlation matrix of Spearman test between the potential factors of EQ-5D-5L utility values of RA patients

| Variable | a | b | c | d | e | f | g | h | i | j | k | l | m | n | o | p | q | r | s | t | u |
|---|---|---|---|---|---|---|---|---|---|---|---|---|---|---|---|---|---|---|---|---|---|
| **b** | -0.148 | | | | | | | | | | | | | | | | | | | | |
| **c** | -0.152 | 0.906 | | | | | | | | | | | | | | | | | | | |
| **d** | -0.031 | 0.384 | 0.174 | | | | | | | | | | | | | | | | | | |
| **e** | -0.220 | 0.285 | 0.423 | 0.509 | | | | | | | | | | | | | | | | | |
| **f** | -0.226 | 0.623 | 0.661 | 0.085 | 0.210 | | | | | | | | | | | | | | | | |
| **g** | -0.143 | 0.628 | 0.662 | 0.212 | 0.279 | 0.629 | | | | | | | | | | | | | | | |
| **h** | -0.445 | 0.334 | 0.142 | 0.327 | 0.253 | 0.173 | 0.144 | | | | | | | | | | | | | | |
| **i** | -0.450 | 0.370 | 0.215 | 0.392 | 0.382 | 0.242 | 0.188 | 0.851 | | | | | | | | | | | | | |
| **j** | -0.255 | 0.334 | 0.274 | 0.335 | 0.307 | 0.295 | 0.318 | 0.551 | 0.573 | | | | | | | | | | | | |
| **k** | -0.314 | -0.087 | 0.051 | -0.183 | 0.227 | 0.031 | -0.003 | -0.045 | -0.027 | 0.024 | | | | | | | | | | | |
| **l** | -0.167 | 0.057 | 0.118 | -0.036 | 0.166 | 0.098 | -0.006 | 0.012 | 0.113 | 0.056 | 0.005 | | | | | | | | | | |
| **m** | -0.146 | 0.108 | 0.157 | -0.149 | 0.040 | 0.161 | 0.117 | 0.149 | 0.090 | 0.038 | -0.094 | 0.164 | | | | | | | | | |
| **n** | 0.278 | -0.054 | -0.165 | 0.110 | -0.248 | -0.153 | -0.043 | -0.080 | -0.169 | -0.158 | -0.148 | -0.713 | -0.299 | | | | | | | | |
| **o** | -0.134 | 0.135 | 0.205 | 0.237 | 0.461 | 0.290 | 0.314 | 0.276 | 0.254 | 0.274 | -0.086 | 0.076 | 0.094 | -0.120 | | | | | | | |
| **p** | 0.206 | 0.013 | 0.000 | -0.115 | -0.120 | -0.144 | -0.079 | -0.026 | 0.042 | -0.069 | -0.043 | -0.123 | -0.069 | 0.105 | -0.261 | | | | | | |
| **q** | -0.163 | -0.001 | 0.046 | -0.051 | 0.131 | 0.040 | -0.041 | 0.030 | 0.157 | 0.042 | 0.039 | 0.793 | 0.090 | -0.564 | -0.002 | -0.075 | | | | | |
| **r** | -0.311 | 0.015 | -0.071 | 0.240 | 0.034 | 0.132 | 0.034 | 0.123 | 0.187 | 0.176 | -0.061 | 0.006 | -0.116 | -0.002 | 0.032 | -0.182 | -0.024 | | | | |
| **s** | 0.043 | -0.227 | -0.233 | -0.048 | 0.025 | -0.036 | -0.265 | 0.062 | 0.037 | -0.036 | 0.197 | 0.020 | -0.006 | -0.040 | -0.032 | 0.077 | -0.034 | -0.029 | | | |
| **t** | 0.131 | -0.161 | -0.192 | -0.001 | -0.102 | -0.168 | -0.041 | -0.099 | -0.133 | -0.129 | 0.063 | -0.864 | -0.180 | 0.717 | -0.079 | 0.114 | -0.603 | 0.014 | -0.040 | | |
| **u** | -0.183 | 0.069 | 0.149 | -0.142 | 0.175 | 0.130 | 0.099 | 0.075 | 0.124 | 0.100 | -0.054 | 0.417 | 0.367 | -0.561 | 0.124 | -0.099 | 0.332 | -0.067 | -0.023 | -0.365 | |
| **v** | -0.148 | -0.002 | -0.025 | -0.059 | -0.150 | 0.061 | -0.019 | 0.033 | 0.045 | 0.005 | -0.132 | -0.059 | 0.126 | 0.107 | 0.038 | 0.046 | 0.036 | -0.007 | -0.043 | 0.040 | -0.002 |

Note： a: EQ-5D-5L score; b: DAS28-CRP; c: DAS28-ESR; d: CRP; e: ESR; f: TJC; g: SJC; h: PtAAP-VAS; i: PtGADA-VAS; j: PhGADA-VAS; k: Gender; l: Habitation; m: Marriage; n: Education; o: Disease state; p: TM; q: MIT; r: GH; s: Ethnicity; t: Occupation; u: Age

DAS28: disease activity scores including 28 joint counts; ESR: erythrocyte sedimentation rate; CRP: high-sensitivity C-reactive protein; TJC: tender joints count; SJC: swollen joints count; PtAAP-VAS: patient's assessment of arthritis pain; PtGADA-VAS: patient's global assessment of disease activity; PhGADA-VAS: physician’s global assessment of disease activity; MIT: Medical insurance type; TM: Treatment methods; GH: General health.

**Appendix 2 Characteristics, patient-/clinician-reported outcomes and associated EQ-5D-5L utility values of RA patients**

| Items | n (%)/Mean (SD) | Mean HUV (SD)/CHUV | Range | | *p* Value | 95% CI | |
|---|---|---|---|---|---|---|---|
| | | | Min | Max | | | |
| **Characteristics** | | **Mean HUV (SD)** | | | | | |
| **Total** | 171(100.0%) | 0.586(0.279) | -0.193 | 1.000 | | 0.544 | 0.629 |
| **Total (adjusted)** | | 0.561 | | | | | |
| **Gender** | | | | | 0.0001** | | |
| Male | 60(35.1%) | 0.697(0.233) | 0.050 | 1.000 | | 0.637 | 0.758 |
| Female | 111(64.9%) | 0.527(0.285) | -0.193 | 1.000 | | 0.473 | 0.580 |
| **Age (years)** | | | | | 0.0008** | | |
| 18-39 | 32(18.7%) | 0.738(0.227) | -0.056 | 1.000 | | 0.656 | 0.819 |
| 40-49 | 35(20.5%) | 0.596(0.234) | 0.052 | 0.942 | | 0.516 | 0.677 |
| 50-59 | 58(33.9%) | 0.513(0.284) | -0.193 | 0.942 | | 0.438 | 0.587 |
| 60-70 | 46(26.9%) | 0.567(0.304) | -0.121 | 1.000 | | 0.476 | 0.657 |
| **Ethnicity** | | | | | 0.3685 | | |
| Han | 162(94.7%) | 0.582(0.282) | -0.193 | 1.000 | | 0.538 | 0.625 |
| Other | 9(5.3%) | 0.673(0.212) | 0.251 | 0.942 | | 0.510 | 0.836 |
| **BMI** | | | | | 0.0606 | | |
| BMI＜18.5 | 19(11.1%) | 0.700(0.185) | 0.147 | 1.000 | | 0.611 | 0.789 |
| 18.5≤BMI＜24 | 95(55.6%) | 0.601(0.274) | -0.100 | 0.955 | | 0.545 | 0.657 |
| 24≤BMI | 57(33.3%) | 0.525(0.302) | -0.193 | 1.000 | | 0.445 | 0.605 |
| **Habitation** | | | | | 0.0158* | | |
| Urban | 75(43.9%) | 0.639(0.274) | -0.193 | 1.000 | | 0.576 | 0.703 |
| Rural | 96(56.1%) | 0.545(0.278) | -0.121 | 1.000 | | 0.489 | 0.601 |
| **Marriage** | | | | | 0.0255* | | |
| Unmarried | 12(7.0%) | 0.740(0.198) | 0.288 | 0.952 | | 0.614 | 0.865 |
| Married | 94(55.0%) | 0.606(0.293) | -0.193 | 1.000 | | 0.546 | 0.666 |
| Divorce/Widowed | 13(7.6%) | 0.571(0.282) | 0.095 | 1.000 | | 0.401 | 0.742 |
| Not reported | 52(30.4%) | 0.520(0.256) | -0.121 | 0.942 | | 0.449 | 0.592 |
| **Occupation** | | | | | 0.1703 | | |
| Farmer | 96(56.1%) | 0.547(0.280) | -0.121 | 1.000 | | 0.491 | 0.604 |
| Worker | 40(23.4%) | 0.648(0.280) | -0.193 | 0.952 | | 0.558 | 0.737 |
| Government-affiliated institutions | 7(4.1%) | 0.727(0.198) | 0.451 | 1.000 | | 0.543 | 0.910 |

| | | | | | | | |
|---|---|---|---|---|---|---|---|
| Retiree | 7(4.1%) | 0.525(0.396) | -0.100 | 0.942 | | 0.159 | 0.891 |
| Other | 21(12.3%) | 0.623(0.235) | 0.102 | 0.952 | | 0.516 | 0.730 |
| **Education** | | | | | 0.0005** | | |
| Primary school or below | 74(43.3%) | 0.508(0.290) | -0.121 | 1.000 | | 0.441 | 0.575 |
| Middle school | 67(39.2%) | 0.608(0.263) | -0.193 | 0.952 | | 0.544 | 0.672 |
| Undergraduate or above | 30(17.5%) | 0.732(0.224) | -0.056 | 1.000 | | 0.648 | 0.815 |
| **Person's annual income** | | | | | 0.2277 | | |
| (0,30000) | 31(18.1%) | 0.556(0.280) | -0.121 | 0.942 | | 0.453 | 0.659 |
| [30000,60000) | 17(9.9%) | 0.675(0.284) | -0.100 | 0.942 | | 0.529 | 0.821 |
| ≥60000 | 16(9.4%) | 0.647(0.300) | -0.193 | 0.942 | | 0.487 | 0.807 |
| Not reported | 107(62.6%) | 0.572(0.275) | -0.067 | 1.000 | | 0.519 | 0.625 |
| **Medical insurance type** | | | | | 0.0099** | | |
| BMIUE | 46(26.9%) | 0.661(0.298) | -0.193 | 1.000 | | 0.572 | 0.749 |
| BMIURR | 73(42.7%) | 0.587(0.275) | -0.067 | 1.000 | | 0.523 | 0.651 |
| Not reported | 52(30.4%) | 0.520(0.256) | -0.121 | 0.942 | | 0.449 | 0.592 |
| **General health** | | | | | 0.0001** | | |
| Good | 3(1.8%) | 0.781(0.099) | 0.703 | 0.893 | | 0.534 | 1.028 |
| General | 131(76.6%) | 0.634(0.261) | -0.121 | 1.000 | | 0.589 | 0.679 |
| Bad | 26(15.2%) | 0.449(0.287) | -0.193 | 0.903 | | 0.333 | 0.565 |
| Very bad | 11(6.4%) | 0.294(0.219) | 0.034 | 0.766 | | 0.147 | 0.441 |
| **Disease stage** | | | | | 0.0331* | | |
| Mid-term | 146(85.4%) | 0.612(0.259) | -0.193 | 1.000 | | 0.568 | 0.654 |
| Serious | 25(14.6%) | 0.440(0.350) | -0.100 | 0.955 | | 0.295 | 0.584 |
| **Treatment methods** | | | | | 0.0230* | | |
| Hospitalization | 16(9.4%) | 0.449(0.246) | 0.101 | 0.766 | | 0.318 | 0.580 |
| Outpatient | 155(90.6%) | 0.601(0.279) | -0.193 | 1.000 | | 0.556 | 0.645 |
| **Das28-CRP** | | | | | 0.6581 | | |
| Remission | 3(1.75%) | 0.744(0.026) | 0.724 | 0.773 | | 0.679 | 0.808 |
| Low | 3(1.75%) | 0.556(0.417) | 0.095 | 0.907 | | -0.480 | 1.591 |
| Moderate | 54(31.58%) | 0.606(0.281) | -0.100 | 0.952 | | 0.529 | 0.683 |
| High | 111(64.91%) | 0.574(0.280) | -0.193 | 1.000 | | 0.521 | 0.626 |
| **Das28-ESR** | | | | | 0.1508 | | |
| Remission | 4(2.34%) | 0.766(0.127) | 0.665 | 0.942 | | 0.564 | 0.967 |
| Low | 6(3.51%) | 0.589(0.333) | 0.095 | 0.942 | | 0.239 | 0.938 |
| Moderate | 41(23.98%) | 0.658(0.245) | 0.033 | 0.952 | | 0.581 | 0.736 |

| | | | | | | | |
|---|---|---|---|---|---|---|---|
| High | 120(70.18%) | 0.556(0.287) | -0.193 | 1.000 | | 0.504 | 0.608 |
| | | | | | | | |
| **Patient-reported Outcomes** | | **CHUV** | | | | | |
| PtAAP-VAS | 63.92(18.20) | -0.0062 | 7.00 | 100.00 | 0.000** | -0.0084 | -0.0041 |
| PtGADA-VAS | 64.49(19.23) | -0.0061 | 8.00 | 100.00 | 0.000** | -0.0082 | -0.0041 |
| | | | | | | | |
| **Clinician-reported Outcomes** | **MIS (SD)** | **CHUV** | | | | | |
| ESR | 48.89 (29.26) | -0.0028 | 2.00 | 121.00 | 0.000** | -0.0042 | -0.0014 |
| CRP | 30.60 (36.40) | -0.0009 | 0.50 | 177.10 | 0.138 | -0.0021 | 0.0003 |
| SJC | 14.40 (9.12) | -0.0043 | 2.00 | 54.00 | 0.067 | -0.0090 | 0.0003 |
| TJC | 23.51 (14.53) | -0.0024 | 5.00 | 68.00 | 0.113 | -0.0053 | 0.0006 |
| DAS28-ESR | 5.58 (1.36) | -0.0482 | 1.77 | 9.09 | 0.002** | -0.0788 | -0.0176 |
| DAS29-CRP | 5.46 (1.20) | -0.0453 | 2.01 | 8.32 | 0.011* | -0.0803 | -0.0104 |
| PhGADA-VAS | 63.04 (14.22) | -0.0052 | 21.50 | 97.00 | 0.000** | -0.0081 | -0.0023 |

Note: *$p$<0.05, **$p$<0.01; SD: standard deviation; CHUV: coefficient of health utility value; MDEUS: median EQ-5D-5L utility score; CI: Confidence Interval; BMIUE: Basic medical insurance for urban employees; BMIURR: Basic medical insurance for urban and rural residents; PtAAP-VAS: patient's assessment of arthritis pain; PtGADA-VAS: patient's global assessment of disease activity; PhGADA-VAS: physician’s global assessment of disease activity; ESR: erythrocyte sedimentation rate; CRP: high-sensitivity C-reactive protein; SJC: swollen joints count; TJC: tender joints count; DAS28: disease activity scores including 28 joint counts; MIS: mean index score.

**Appendix 3 The proportion of reporting problems on EQ-5D-5L dimensions of RA patients with various characteristics**

| Characteristics | Mobility | | Self-care | | Usual activities | | Pain/Discomfort | | Anxiety/Depression | |
|---|---|---|---|---|---|---|---|---|---|---|
| | % With Problems | *p* Value | % With Problems | *p* Value | % With Problems | *p* Value | % With Problems | *p* Value | % With Problems | *p* Value |
| **Total** | 76.02% | | 72.51% | | 74.85% | | 84.21% | | 64.33% | |
| **Total (Adjust)** | 80.91% | | 76.59% | | 81.02% | | 83.64% | | 65.33% | |
| **Gender** | | 0.0005** | | 0.0036** | | 0.0001** | | 0.6834 | | 0.4724 |
| Male | 55.00% | | 55.00% | | 48.33% | | 86.67% | | 60.00% | |
| Female | 87.39% | | 81.98% | | 89.19% | | 82.88% | | 66.67% | |
| **Age** | | 0.8703 | | 0.9758 | | 0.5351 | | 0.0232* | | 0.1201 |
| 18-39 | 68.75% | | 75.00% | | 62.50% | | 56.25% | | 46.88% | |
| 40-49 | 80.00% | | 74.29% | | 77.14% | | 94.29% | | 68.57% | |
| 50-59 | 77.59% | | 72.41% | | 81.03% | | 91.38% | | 75.86% | |
| 60-70 | 76.09% | | 69.57% | | 73.91% | | 86.96% | | 58.70% | |
| **Ethnicity** | | 0.9256 | | 0.7556 | | 0.8763 | | 0.4006 | | 0.2899 |
| Han | 75.93% | | 72.84% | | 74.69% | | 83.33% | | 65.43% | |
| Other | 77.78% | | 66.67% | | 77.78% | | 100.00% | | 44.44% | |
| **BMI** | | 0.5527 | | 0.1676 | | 0.4530 | | 0.2216 | | 0.1332 |
| BMI＜18.5 | 68.42% | | 94.74% | | 89.47% | | 63.16% | | 47.37% | |
| 18.5≤BMI＜24 | 73.68% | | 67.37% | | 74.74% | | 85.26% | | 61.05% | |
| 24≤BMI | 82.46% | | 73.68% | | 70.18% | | 89.47% | | 75.44% | |
| **Habitation** | | 0.1817 | | 0.0892 | | 0.1713 | | 0.4006 | | 0.9478 |
| Urban | 69.33% | | 64.00% | | 68.00% | | 80.00% | | 64.00% | |
| Rural | 81.25% | | 79.17% | | 80.21% | | 87.50% | | 64.58% | |
| **Marriage** | | 0.2717 | | 0.6089 | | 0.2202 | | 0.0110* | | 0.3020 |
| Unmarried | 66.67% | | 91.67% | | 66.67% | | 41.67% | | 58.33% | |
| Married | 72.34% | | 71.28% | | 68.09% | | 79.79% | | 59.57% | |
| Divorce/ Widowed | 61.54% | | 61.54% | | 76.92% | | 92.31% | | 53.85% | |
| Not reported | 88.46% | | 73.08% | | 88.46% | | 100.00% | | 76.92% | |
| **Occupation** | | 0.8855 | | 0.4108 | | 0.6206 | | 0.3779 | | 0.9916 |
| Farmer | 80.21% | | 78.13% | | 79.17% | | 88.54% | | 63.54% | |
| Worker | 70.00% | | 57.50% | | 62.50% | | 82.50% | | 65.00% | |
| Government-affiliated institutions | 71.43% | | 85.71% | | 71.43% | | 42.86% | | 57.14% | |

| | | | | | | | | | | |
|---|---|---|---|---|---|---|---|---|---|---|
| Retiree | 71.43% | | 71.43% | | 71.43% | | 85.71% | | 71.43% | |
| Other | 71.43% | | 71.43% | | 80.95% | | 80.95% | | 66.67% | |
| **Education** | | 0.3952 | | 0.3328 | | 0.0166* | | 0.1792 | | 0.8839 |
| Primary school or below | 82.43% | | 79.73% | | 89.19% | | 86.49% | | 66.22% | |
| Middle school | 73.13% | | 68.66% | | 65.67% | | 89.55% | | 64.18% | |
| Undergraduate or above | 66.67% | | 63.33% | | 60.00% | | 66.67% | | 60.00% | |
| **Person's annual income** | | 0.3404 | | 0.9521 | | 0.3767 | | 0.2002 | | 0.5122 |
| (0,30000) | 83.87% | | 77.42% | | 77.42% | | 100.00% | | 64.52% | |
| [30000,60000) | 52.94% | | 70.59% | | 52.94% | | 64.71% | | 64.71% | |
| [60000,+∞） | 75.00% | | 75.00% | | 68.75% | | 75.00% | | 50.00% | |
| Not reported | 77.57% | | 71.03% | | 78.50% | | 84.11% | | 67.29% | |
| **Medical insurance type** | | 0.1591 | | 0.2330 | | 0.0396* | | 0.0546 | | 0.1629 |
| BMIUE | 67.39% | | 60.87% | | 58.70% | | 73.91% | | 60.87% | |
| BMIURR | 72.60% | | 79.45% | | 75.34% | | 79.45% | | 57.53% | |
| Not reported | 88.46% | | 73.08% | | 88.46% | | 100.00% | | 76.92% | |
| **Health status** | | 0.1765 | | 0.3881 | | 0.2407 | | 0.9358 | | 0.0205* |
| Good | 66.67% | | 66.67% | | 33.33% | | 100.00% | | 33.33% | |
| General | 70.99% | | 69.47% | | 71.76% | | 83.21% | | 58.02% | |
| Bad | 96.15% | | 76.92% | | 88.46% | | 88.46% | | 84.62% | |
| Very bad | 90.91% | | 100.00% | | 90.91% | | 81.82% | | 100.00% | |
| **Disease stage** | | 0.4560 | | 0.4842 | | 0.2192 | | 0.6940 | | 0.0660 |
| Middle | 74.66% | | 71.23% | | 72.60% | | 84.93% | | 60.96% | |
| Advanced | 84.00% | | 80.00% | | 88.00% | | 80.00% | | 84.00% | |
| **Das28-CRP** | | | | | | | | | | |
| Remission | | 0.6509 | | 0.3498 | | 0.0662 | | 0.0733 | | 0.9744 |
| Low | 100.00% | | 100.00% | | 100.00% | | 33.33% | | 66.67% | |
| Moderate | 66.67% | | 100.00% | | 100.00% | | 66.67% | | 66.67% | |
| High | 72.22% | | 66.67% | | 62.96% | | 83.33% | | 66.67% | |
| **Das28-ESR** | 77.48% | | 73.87% | | 79.28% | | 86.49% | | 63.06% | |
| Remission | | | | | | | | | | |
| Low | | 0.0592 | | 0.5956 | | 0.0049** | | 0.0733 | | 0.1925 |
| Moderate | 75.00% | | 50.00% | | 75.00% | | 75.00% | | 25.00% | |
| High | 66.67% | | 83.33% | | 83.33% | | 83.33% | | 66.67% | |
| **Treatment methods** | | 0.4050 | | 0.5262 | | 0.9919 | | 0.8114 | | 0.4387 |
| Hospitalization | 87.50% | | 81.25% | | 75.00% | | 87.50% | | 75.00% | |

| | | | | | |
|---|---|---|---|---|---|
| Outpatient | 74.84% | 71.61% | 74.84% | 83.87% | 63.23% |

Note：*$p$<0.05, **$p$<0.01; BMIUE: Basic medical insurance for urban employees; BMIURR: Basic medical insurance for urban and rural residents.

## Appendix 4 EQ-VAS score of RA patients

| EQ-VAS | Mean (SD) | Median | Range | | 95% Cl | |
|---|---|---|---|---|---|---|
| | | | Min | Max | | |
| female | 47.22(19.75) | 49.00 | 10.00 | 90.00 | 43.50 | 50.93 |
| male | 47.41(19.59） | 45.00 | 10.00 | 90.00 | 42.34 | 52.47 |
| Total | 47.28(19.64) | 48.00 | 10.00 | 90.00 | 44.32 | 50.25 |
| Total(adjusted) | 47.25 | | | | | |

Note: SD: Standard deviation; CI: Confidence interval.

## Appendix 5 VIF of variables included in the regression model

| Variables | | VIF | 1/VIF |
|---|---|---|---|
| **Age** | 18-39 | | |
| | 40-49 | 1.76 | 0.568 |
| | 50-59 | 2.28 | 0.439 |
| | 60-70 | 2.18 | 0.460 |
| **Gender** | Male | | |
| | Female | 1.14 | 0.881 |
| **BMI** | BMI<18.5 | | |
| | 18.5≤BMI<24 | 2.95 | 0.339 |
| | 24≤BMI | 2.95 | 0.339 |
| **Habitation** | Urban | | |
| | Rural | 1.21 | 0.824 |
| **Disease stage** | Mid-term | | |
| | Serious | 1.17 | 0.851 |
| **Treatment methods** | Hospitalization | | |
| | Outpatient | 1.13 | 0.888 |
| **PtGADA-VAS** | | 1.16 | 0.863 |
| **DAS28-ESR** | | 1.13 | 0.882 |
| **Mean VIF** | | 1.73 | |

Note：VIF: variance inflation factor.

**Appendix 6 Questionnaires on the Quality of Life of Chinese RA Patients (Chinese to English version)**

Survey ID：□□ □□ □□ □□□ □

Interviewer：________________ Interviewer ID：□□

Survey Date：_______________

# No.1 Questionnaires for Patient

**EQ-5D-5L**

**Under each heading, please tick the ONE box that best describes your health TODAY.**

**MOBILITY**

I have no problems in walking about ❑

I have slight problems in walking about ❑

I have moderate problems in walking about ❑

I have severe problems in walking about ❑

I am unable to walk about ❑

**SELF-CARE**

I have no problems washing or dressing myself ❑

I have slight problems washing or dressing myself ❑

I have moderate problems washing or dressing myself ❑

I have severe problems washing or dressing myself ❑

I am unable to wash or dress myself ❑

**USUAL ACTIVITIES** (e.g. work, study, housework, family or leisure activities)

I have no problems doing my usual activities ❑

I have slight problems doing my usual activities ❑

I have moderate problems doing my usual activities ❑

I have severe problems doing my usual activities ❑

I am unable to do my usual activities ❑

**PAIN / DISCOMFORT**

I have no pain or discomfort ❑

I have slight pain or discomfort ❑

I have moderate pain or discomfort ❑

I have severe pain or discomfort ❑

I have extreme pain or discomfort ❑

**ANXIETY / DEPRESSION**

I am not anxious or depressed ❑

I am slightly anxious or depressed ❑

I am moderately anxious or depressed ❑

I am severely anxious or depressed ❑

I am extremely anxious or depressed ❑

We would like to know how good or bad your health is TODAY.

This scale is numbered from 0 to 100.

100 means the best health you can imagine.

0 means the worst health you can imagine.

Mark an X on the scale to indicate how your health is TODAY.

Now, please write the number you marked on the scale in the box below.

YOUR HEALTH TODAY = ☐

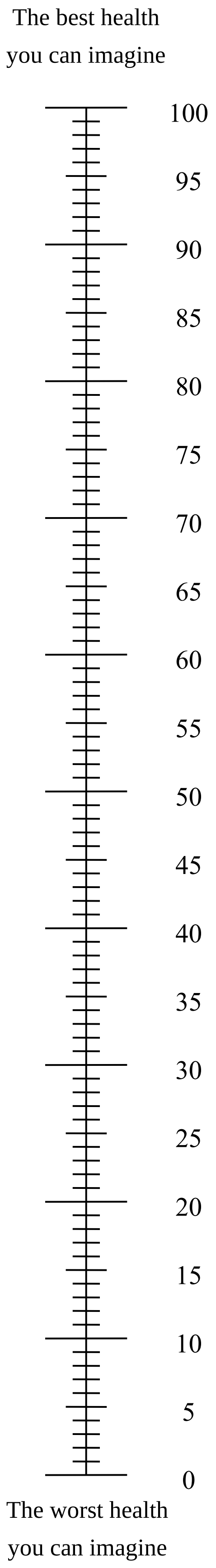

**The patient's assessment of arthritis pain visual analogue scale (PtAAP-VAS)**

Please mark a vertical line on the scale below to indicate the level of pain you are experiencing due to arthritis TODAY.

No pain　　　　　　　　　　　　　　Most severe pain

0　　　　　　　　　　　　　　　　　100

**The patient's global assessment of disease activity visual analogue scale (PtGADA-VAS)**

Please consider the impact of arthritis on all aspects of your life, then mark a vertical line on the scale below to indicate how you feel TODAY.

Very good/No symptoms　　　　　　　Very bad / Severe symptoms

0　　　　　　　　　　　　　　　　　100

**Patients' demographic characteristics**

Here are some questions about your basic information. Please put a check mark "√" next to the options that apply to your personal situation, and fill in the blank with the information you need to provide. Note: This is an anonymous survey, your personal information will not be disclosed, and there are no right or wrong answers to your responses.

| | |
|---|---|
| 1.What is your gender? | ❑Male ❑Female |
| 2. What is your age? | _________Year |
| 3. What is your ethnicity? | _________ |
| 4. What are your weight and height respectively? | _________kg _________cm |
| 5. Where do you currently live? | ❑ Rural ❑Urban |
| 6.What is your marital status? | ❑ Unmarried ❑ Married<br>❑ Divorce ❑ Widowed |
| 7. What is your level of education? | ❑ Primary school or below ❑ Middle school ❑ Undergraduate or above |
| 8. What is your Occupation? | ❑ Farmer ❑ Worker<br>❑ Government-affiliated institutions<br>❑ Retiree ❑ Other |
| 9.What is your treatment methods? | ❑ Hospitalization ❑ Outpatient |
| 10. How much is your personal annual income?（ten thousand yuan） | _________ ten thousand yuan |
| 11.What is your medical insurance type? | ❑ Basic medical insurance for urban |

| | |
|---|---|
| | employees<br>❑ Basic medical insurance for urban and rural residents |
| 12. How do you feel about your current health status? | ❑ Good ❑ Genaral<br>❑ Bad ❑ Very bad |

# No.2 Questionnaires for Physician

**The following questions should be completed by the patient's attending physician.**

1.Patient’s disease stage

❑ Stage I (Early stage)
❑ Stage II (Middle stage)
❑ Stage III (Advanced stage)

2.Patient’s erythrocyte sedimentation rate (ESR, unit: mm/h) _________

3.Patient’s high-sensitivity C-reactive protein (CRP unit: mg/L) _________

**The physician’s global assessment of disease activity visual analogue scale (PhGADA-VAS)**

Please mark a vertical line on the scale below to assess the overall condition of signs and symptoms of rheumatoid arthritis in the subjects as well as the functional ability of the subjects.

Very good/No symptoms
No limit for Normal activities

Very bad/Severe and intolerable symptoms
Unable to perform any normal activities

0 ———————————————— 100

**Assessment of swollen joints count and tender joints count**

| Right | | | | | | Joint | Left | | | | | |
|---|---|---|---|---|---|---|---|---|---|---|---|---|
| Tender | | | Swollen | | | | Swollen | | | Tender | | |
| Yes | No | Not done | Yes | No | Not done | | Yes | No | Not done | Yes | No | Not done |
| □ | □ | □ | □ | □ | □ | Temporomandibular Joint | □ | □ | □ | □ | □ | □ |
| □ | □ | □ | □ | □ | □ | Sternoclavicular Joint | □ | □ | □ | □ | □ | □ |
| □ | □ | □ | □ | □ | □ | Acromioclavicular Joint | □ | □ | □ | □ | □ | □ |
| □ | □ | □ | □ | □ | □ | Shoulder Joint | □ | □ | □ | □ | □ | □ |
| □ | □ | □ | □ | □ | □ | Elbow joint | □ | □ | □ | □ | □ | □ |
| □ | □ | □ | □ | □ | □ | Wrist joint | □ | □ | □ | □ | □ | □ |
| □ | □ | □ | □ | □ | □ | First metacarpophalangeal joint | □ | □ | □ | □ | □ | □ |
| □ | □ | □ | □ | □ | □ | Second metacarpophalangeal joint | □ | □ | □ | □ | □ | □ |
| □ | □ | □ | □ | □ | □ | Third metacarpophalangeal joint | □ | □ | □ | □ | □ | □ |
| □ | □ | □ | □ | □ | □ | Fourth metacarpophalangeal joint | □ | □ | □ | □ | □ | □ |
| □ | □ | □ | □ | □ | □ | Fifth metacarpophalangeal joint | □ | □ | □ | □ | □ | □ |
| □ | □ | □ | □ | □ | □ | Thumb interphalangeal joint | □ | □ | □ | □ | □ | □ |
| □ | □ | □ | □ | □ | □ | Proximal interphalangeal joint of the upper limb second digit | □ | □ | □ | □ | □ | □ |
| □ | □ | □ | □ | □ | □ | Proximal interphalangeal joint of the upper limb third digit | □ | □ | □ | □ | □ | □ |
| □ | □ | □ | □ | □ | □ | Proximal interphalangeal joint of the upper limb fourth digit | □ | □ | □ | □ | □ | □ |
| □ | □ | □ | □ | □ | □ | Proximal interphalangeal joint of the upper limb fifth digit | □ | □ | □ | □ | □ | □ |
| □ | □ | □ | □ | □ | □ | Distal interphalangeal joint of the second digit | □ | □ | □ | □ | □ | □ |
| □ | □ | □ | □ | □ | □ | Distal interphalangeal joint of the third digit | □ | □ | □ | □ | □ | □ |
| □ | □ | □ | □ | □ | □ | Distal interphalangeal joint of the fourth digit | □ | □ | □ | □ | □ | □ |
| □ | □ | □ | □ | □ | □ | Distal interphalangeal joint of the fifth digit | □ | □ | □ | □ | □ | □ |

| | | | | | | | | | | | | |
|---|---|---|---|---|---|---|---|---|---|---|---|---|
| □ | □ | □ | | | | Hip joint | □ | □ | □ | | | |
| □ | □ | □ | □ | □ | □ | Knee joint | □ | □ | □ | □ | □ | □ |
| □ | □ | □ | □ | □ | □ | Ankle joint | □ | □ | □ | □ | □ | □ |
| □ | □ | □ | □ | □ | □ | Tarsal bone | □ | □ | □ | □ | □ | □ |
| □ | □ | □ | □ | □ | □ | First metatarsophalangeal joint | □ | □ | □ | □ | □ | □ |
| □ | □ | □ | □ | □ | □ | Second metatarsophalangeal joint | □ | □ | □ | □ | □ | □ |
| □ | □ | □ | □ | □ | □ | Third metatarsophalangeal joint | □ | □ | □ | □ | □ | □ |
| □ | □ | □ | □ | □ | □ | Fourth metatarsophalangeal joint | □ | □ | □ | □ | □ | □ |
| □ | □ | □ | □ | □ | □ | Fifth metatarsophalangeal joint | □ | □ | □ | □ | □ | □ |
| □ | □ | □ | □ | □ | □ | Hallux interphalangeal joint | □ | □ | □ | □ | □ | □ |
| □ | □ | □ | □ | □ | □ | Proximal interphalangeal joint of the second toe | □ | □ | □ | □ | □ | □ |
| □ | □ | □ | □ | □ | □ | Proximal interphalangeal joint of the third toe | □ | □ | □ | □ | □ | □ |
| □ | □ | □ | □ | □ | □ | Proximal interphalangeal joint of the fourth toe | □ | □ | □ | □ | □ | □ |
| □ | □ | □ | □ | □ | □ | Proximal interphalangeal joint of the fifth toe | □ | □ | □ | □ | □ | □ |

Tender joints count: ___/___ Swollen joints count: ___/___
